# High-Performance Liquid Chromatographic and High-Performance Thin-Layer Chromatographic Method for the Quantitative Estimation of Dolutegravir Sodium in Bulk Drug and Pharmaceutical Dosage Form

**DOI:** 10.3797/scipharm.1507-09

**Published:** 2015-08-27

**Authors:** Girija B. Bhavar, Sanjay S. Pekamwar, Kiran B. Aher, Ravindra S. Thorat, Sanjay R. Chaudhari

**Affiliations:** 1 Department of Pharmaceutical Chemistry, Amrutvahini College of Pharmacy, Sangamner – 422608, Maharashtra, India; 2 School of Pharmacy, Swami Ramanand Teerth Marathwada University, Nanded – 431606, Maharashtra, India

**Keywords:** Dolutegravir sodium, HPLC, Forced Degradation, HPTLC, Validation

## Abstract

Simple, sensitive, precise, and specific high-performance liquid chromategraphic (HPLC) and high-performance thin-layer chromatographic (HPTLC) methods for the determination of dolutegravir sodium in bulk drug and pharmaceutical dosage form were developed and validated. In the HPLC method, analysis of the drug was carried out on the ODS C_18_ column (150 × 4.6 mm, 5 μm particle size) using a mixture of acetonitrile: water (pH 7.5) in the ratio of 80:20 v/v as the mobile phase at the flow rate 1 mL/min at 260 nm. This method was found to be linear in the concentration range of 5–35 μg/mL. The peak for dolutegravir sodium was observed at 3.0 ± 0.1 minutes. In the HPTLC method, analysis was performed on aluminum-backed plates pre-coated with silica gel G60 F_254_ using methanol: chloroform: formic acid in the proportion of 8:2:0.5 v/v/v as the mobile phase. This solvent system was found to give compact spots for dolutegravir sodium with the *Rf* value 0.77 ± 0.01. Densitometric analysis of dolutegravir sodium was carried out in the absorbance mode at 265 nm. Linear regression analysis showed good linearity with respect to peak area in the concentration range of 200–900 ng/spot. The methods were validated for precision, limit of detection (LOD), limit of quantitation (LOQ), accuracy, and specificity. Statistical analysis showed that both of the methods are repeatable and specific for the estimation of the said drug. The methods can be used for routine quality control analysis of dolutegravir sodium.

## Introduction

**Fig. 1:**
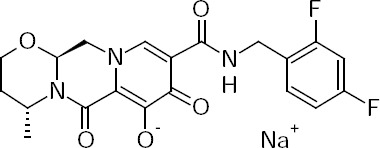
Structure of dolutegravir sodium

Dolutegravir sodium, chemically, sodium (4*R*,12a*S*)-9-[(2,4-difluorobenzyl)carbamoyl]-4-methyl-6,8-dioxo-3,4,6,8,12,12a-hexahydro-2*H*-pyrido[1’,2’:4,5]pyrazino[2,1-*b*][1,3]oxazin-7-olate (Fig. 1), is a novel integrase stand transfer inhibitor active against human immunedeficiency virus. The drug is active against HIV type 1 (HIV-1) and also has some *in vitro* activity against HIV type 2 (HIV-2) [1–4].

The literature review revealed a liquid chromatography–tandem mass spectrometry method [5] and a sensitive HPLC–MS/MS method [6] for the estimation of dolutegravir in human blood plasma. There is no HPLC and HPTLC method available yet for the quantitative determination of dolutegravir in tablet formulation. Further, no official or draft monograph of dolutegravir sodium was published in any of the pharmacopoeia for compendia applications.

The aim of the present work was to develop simple, economic, precise, accurate, specific, stability-indicating HPLC method and a simple HPTLC method for the assay determination of dolutegravir in bulk form and/or in pharmaceutical dosage form. The developed methods were validated as per International Conference on Harmonisation (ICH) guidelines [7–9].

## Experimental

### Chemicals and Reagents

Dolutegravir sodium bulk drug was obtained from Mylan Labs Ltd, (Hyderabad, India), the commercial tablets of dolutegravir sodium were not available in the Indian market; hence, we have prepared immediate release tablets containing dolutegravir sodium equivalent to 50 mg of dolutegravir as per cGMP guidelines. The tablets contain lactose monohydrate, microcrystalline cellulose, starch, and magnesium stearate with an average weight of 250 mg. Water was obtained from a Milli-Q UF-Plus apparatus (Millipore) and was used to prepare all solutions for the method. Other chemicals used were analytical or HPLC-grade and the glassware used was Class A grade.

## Method A: HPLC

### Instrumentation

Chromatographic separation was performed using the Waters 600 Controller chromatographic system equipment with a reciprocating pump UV/Visible detector and Reodyne (7725i) with a 20 μL fixed loop, and the data were analyzed by using Data Ace software. The analytical balance used for weighing the standard and sample was Shimadzu Aux 220 Uni Bloc Pat 1987. The balance (Mettler Toledo XP205, Mumbai) was used for weighing and an Ultrasonicator (Enertech Electronics Pvt. Ltd., Mumbai) was used for sonication. The Thermo Scientific Forma 3960 Series environmental chamber was used for stress testing.

### Chromatographic Conditions

The analysis was performed using the Inertsil ODS C_18_ (150 × 4.6 mm, 5 µm particle size) column using a mobile phase consisting of acetonitrile: water (pH 7.5) in the ratio of 80:20, v/v at a flow rate of 1 mL/min. The eluent was monitored using UV detection at a wavelength of 260 nm at ambient column temperature. The injection volume 20 μL was used. The total run time was 6 min. The mobile phase was filtered through a 0.45 μm micron filter prior to use.

### Preparation of Standard Stock Solution

Accurately weighed dolutegravir sodium standard equivalent to 100 mg dolutegravir was transferred into a 100-mL volumetric flask. It was dissolved in 70 mL of methanol by sonication for one minute and then diluted to volume with methanol to obtain the standard stock solution of the concentration 1000 μg/mL of dolutegravir.

### Selection of Detection Wavelength

The spectra of 10 μg/mL solution of dolutegravir in methanol were recorded on a UV spectrophotometer. The wavelength of the maximum absorbance was observed.

### Preparation of the Calibration Curve

The aliquots from the standard stock solution (0.5, 1, 1.5, 2.0, 2.5, 3.0, and 3.5 mL) were transferred to a series of 100-mL volumetric flasks and diluted up to the mark with mobile phase, to give the working standard solutions of the concentration 5 μg/mL to 35 μg/mL.

### Analysis of the Pharmaceutical Dosage Form

To determine the content of dolutegravir in prepared tablet dosage form, 20 tablets were weighed; their average weight was determined and finely powdered. Powder equivalent to 100 mg dolutegravir was accurately weighed and transferred to a 100-mL volumetric flask and dissolved in 70 mL of methanol and the mixture was sonicated for 10 min. The volume was adjusted up to the mark with the same solvent. This solution was filtered through a 0.45 μm nylon syringe filter. From this solution, an aliquot of 1 mL was further diluted to 100 mL with the mobile phase. The chromatograms were developed using previously described chromatographic conditions. Six replicate injections of this solution were injected and the chromatogram and peak area were recorded. The concentration of the tablet solution was determined using a linear regression equation of a calibration graph and the amount of drug in the tablet was determined. The possibility of excipient interference in the analysis was studied.

### Forced Degradation Studies [8, 9]

The forced degradation studies were performed to establish the stability-indicating nature and specificity of the assay method and to observe any degraded compounds. The forced degradation of dolutegravir sodium sample solution was carried out with 1 N hydrochloric acid, 1 N sodium hydroxide, 30% v/v hydrogen peroxide, UV degradation at 254 nm, and thermal degradation at 60°C in the presence of 80% RH. The forced degradation conditions are as given in Table 1. Chromatograms were recorded for all of the above solutions.

**Tab. 1:**
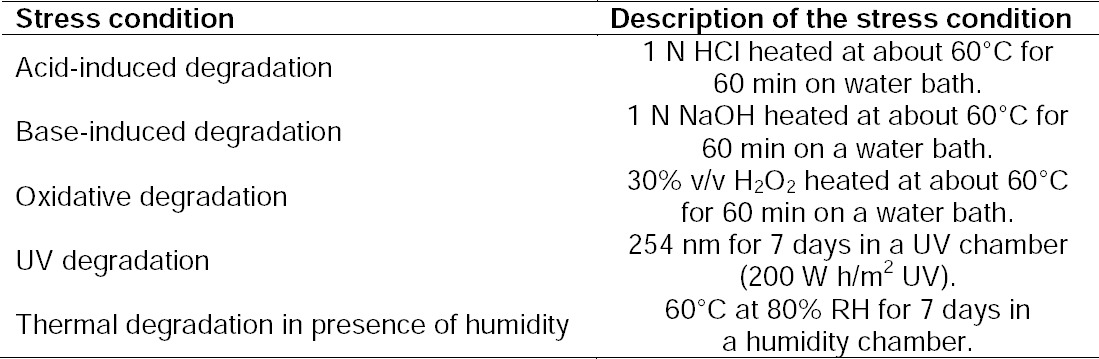
Forced Degradation Conditions

### Method Validation

The HPLC method was validated as per the ICH guidelines [8, 9].

#### Linearity

Twenty μL from each working standard solution was injected in hexaplicate into the HPLC system. The peak areas were plotted against the corresponding concentrations to obtain the calibration graph. A linear calibration curve was generated using least-squares linear regression analysis.

#### Precision

Precision of the method was verified by repeatability and intermediate precision studies. Repeatability studies were performed by analyzing the solution of 20 µg/mL of the drug in hexaplicate on the same day. The % RSD of the six determinations was calculated. Intermediate precision of the method was checked by repeating studies on two different days. The % RSD of 12 determinations was calculated.

#### Accuracy

Accuracy of the method was determined by the standard addition method in which the known amounts of standard dolutegravir solutions were added to a pre-analyzed solution. These amounts corresponded to 80, 100, and 120% of the sample concentration. The amounts of dolutegravir were estimated by applying these values to the regression equation of the calibration curve. An accuracy study was performed for three times at each level, and the % recovery of dolutegravir was calculated.

#### Robustness

To determine the robustness of the method, the experimental conditions, i.e. flow rate and pH of the mobile phase, were deliberately altered. For changes in conditions, the sample was assayed in triplicate. The optimum mobile phase flow rate was 1.0 mL/min. This was changed by 0.1 units to 0.9 and 1.1 mL/min and the effects were studied. The effect of a change in pH of the mobile phase was studied at pH 7.3 and pH 7.7.

#### Limit of Detection and Limit of Quantitation

The LOD and LOQ were calculated by using the formula, LOD = 3.3 × σ/S and LOQ = 10 × σ/S, where σ is a residual standard deviation of the regression line and S is the slope of the corresponding regression line.

#### Specificity

The specificity of the developed method was established by analyzing the sample solutions containing dolutegravir standard and prepared tablets in relation to interferences from formulation ingredients.

#### Solution Stability [10]

The stability of standard solutions was tested after 1, 6, and 24 h of storage. The stability of the solutions was determined by analyzing the sample solution of the concentration 10 µg/mL.

#### System Suitability Parameters

Six replicate injections of system suitability solutions (working standard solution) were injected. The retention time, areas, theoretical plates, peak asymmetry, and resolution were calculated for standard solutions.

## Method B: HPTLC

### HPTLC Instrumentation and Chromatographic Conditions

The HPTLC plates were prewashed with methanol and activated at 110°C for 5 minutes prior to chromatography. The samples were spotted in the form of bands of 8 mm width with a Camag 100 microliter sample syringe (Hamilton, Bonaduz, Switzerland) on HPTLC aluminum-backed plates pre-coated with silica gel G60 F_254_, [(20 *×* 10 cm); E. Merck, Darmstadt, Germany, supplied by Anchrom Technologists, Mumbai] using a Camag Linomat V applicator (Switzerland). Linear ascending development was carried out in a 20 cm *×* 10 cm twin trough glass chamber (Camag, Muttenz, Switzerland) saturated with the mobile phase. The mobile phase consisted of methanol, chloroform, and formic acid in the proportion of 8:2:0.5, v/v/v and 20 mL mobile phase was used per chromatography run. The optimized chamber saturation time for the mobile phase was 30 min using saturation pads at room temperature (25 *±* 2°C). The length of the chromatogram run was 8 cm. Densitometric scanning was performed using a Camag TLC Scanner III in the absorbance mode and operated by WinCATS software (V 3.15, Camag). The slit dimension was kept at 5 mm *×* 0.45 mm and the scanning speed was 10 mm/s. The source of radiation used was a deuterium lamp emitting a continuous UV spectrum between 190 and 400 nm. All determinations were performed at ambient temperature with a detection wavelength of 265 nm.

### Preparation of Standard Solution

Dolutegravir sodium (equivalent to 100 mg of dolutegravir) was accurately weighed and transferred into 100-mL volumetric flasks, and dissolved in 50 mL of methanol and the solution was further diluted with methanol to get the final concentration of 1000 μg/mL. From this stock solution, 10 mL was diluted to 100 mL with methanol to give the final concentration of 100 μg/mL.

### Preparation of Sample Solution

Twenty tablets (each tablet containing dolutegravir 50 mg) were accurately weighed; their average weight was calculated and finely powdered. The powder equivalent to 50 mg dolutegravir was accurately weighed and transferred into 50-mL volumetric flasks containing 20 mL of methanol and ultrasonicated for 15 minutes. The solution was diluted to 50 mL with methanol. The above solution was filtered through the Whatman no. 41 filter paper. From this solution, 5 mL of solution was transferred to 50-mL volumetric flasks and made up to the mark with methanol.

### Analysis of Pharmaceutical Dosage Form

An aliquot of 4 μL was applied on the TLC plates followed by the development and measured at 265 nm. The analysis was repeated six times. The concentration of the sample was determined using the linear regression equation of the calibration graph and the amount of drug in each tablet was determined.

### Method Validation

The HPTLC method was validated as per the ICH guidelines [8–10].

#### Linearity

Aliquots from the standard solution were spotted on the HPTLC plate to obtain concentrations in the range of 200–900 ng/spot. The plate was developed using the previously described mobile phase and scanned. The peak areas were plotted against the corresponding concentrations to obtain the calibration graph. A linear calibration curve was generated using least-squares linear regression analysis.

#### Precision

Precision of the method was verified by repeatability and intermediate precision studies. Repeatability studies were performed by analyses of the drug (400 ng/spot) in hexaplicate on the same day. Intermediate precision of the method was checked by repeating studies on two different days.

#### Limit of Detection and Limit of Quantitation

The sensitivity of the method was determined in terms of limit of detection (LOD) and limit of quantitation (LOQ). The LOD and LOQ were calculated by using the formula, LOD = 3.3 × σ/S and LOQ = 10 × σ/S, where σ is the residual standard deviation of the regression line and S is the slope of the corresponding regression line.

#### Accuracy

The accuracy of the method was determined by the standard addition method in which the known amount of standard dolutegravir solutions were added to pre-analyzed sample solutions. These amounts corresponded to 80, 100, and 120% of the sample concentration. The amounts of dolutegravir were estimated by applying these values to the regression equation of the calibration curve. The accuracy study was performed in triplicate and the % recovery of dolutegravir was calculated.

#### Specificity

The specificity of the developed method was established by comparing the chromatogram of the sample with the chromatograms of the standard.

#### Solution Stability

The stability of the standard solutions was tested after 1, 6, and 24 h of storage. The stability of the solutions was determined by comparing the peak area percentage and peak purity at 500 ng/spot.

## Results and Discussion Method A: HPLC

### Selection of Detection Wavelength

**Fig. 2:**
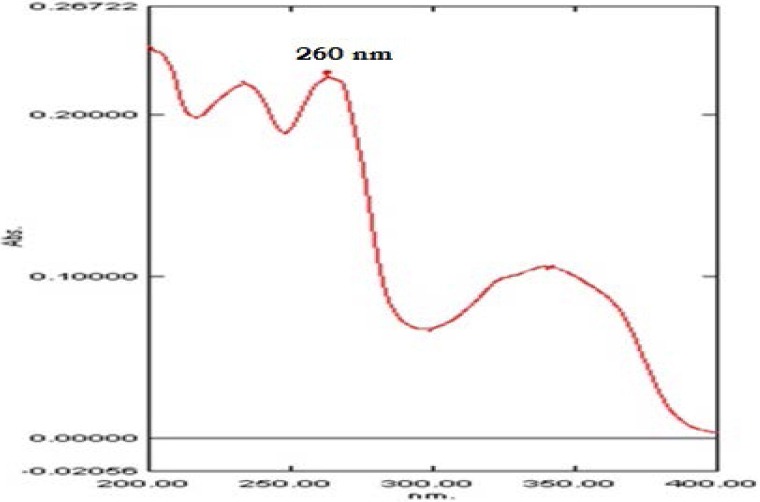
UV spectrum of dolutegravir in methanol

The spectra of dolutegravir showed the maximum absorbance at the wavelength 260.0 nm (Figure 2). So, the wavelength 260.0 nm was selected for the analysis.

### Assay of Dolutegravir in Tablets by the HPTLC Method

The concentration of the tablet solution was determined using a linear regression equation (using the slope and Y-intercept) and the amount of drug in each tablet was determined. The results of the assay of the tablets are summarized in Table 2.

**Tab. 2:**
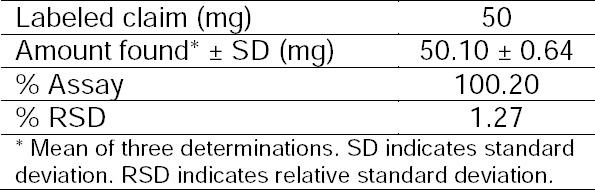
Results of the Assay of Dolutegravir in Tablets by the HPLC Method

### Forced Degradation Studies of Dolutegravir

**Fig. 3:**
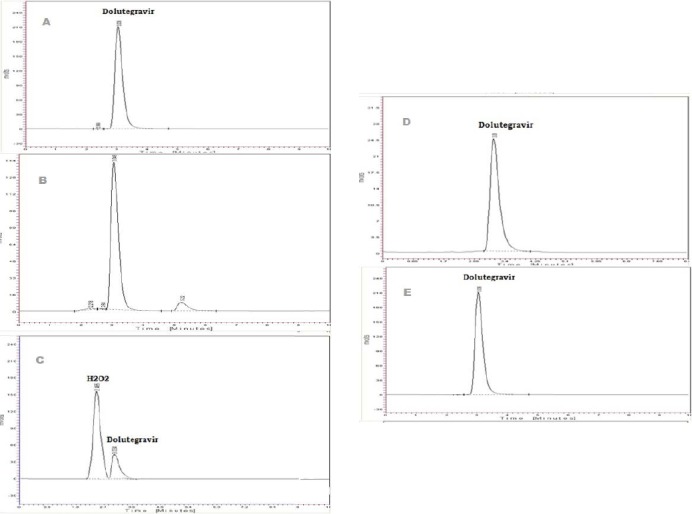
Typical degradation chromatogram of dolutegravir sodium: A- acid-induced degradation; B- alkali-induced degradation; C- peroxide degradation; D- UV degradation; and E- thermal with humidity-induced degradation

The purity of dolutegravir was unaffected by the presence of its degradation products; thus, the method can be said to be stability-indicating. The percent assay of all the degraded samples varied between 92.1 and 100.2%. The numbers of the degradation peaks observed in different stress conditions are shown in Figure 3 and are as follows:

#### Acid-Induced Degradation

Only minor degradation products were observed at Rt 2.36 (0.07%) in the acid degradation of sample preparation.

#### Base-Induced Degradation

Major degradation products were observed at Rt 2.2, 2.6, and 5.6 (9%) in 1 N sodium hydroxide for 80°C for 20 h in the sample preparation.

#### Oxidative Degradation

No degradation peak was observed in the peroxide degradation of the sample preparation.

#### UV Radiation Degradation (at 254 nm)

No degradation product was observed after exposure of the sample to UV light at 254 nm.

#### Thermal with Humidity Degradation (60°C/80% RH)

No degradation peak was observed in the sample preparation exposed to thermal with humidity degradation.

### Method Validation

#### Linearity

**Fig. 4:**
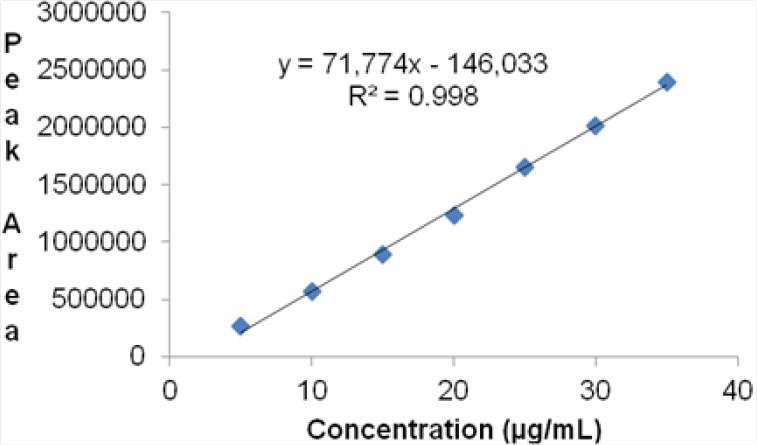
Linearity plot of dolutegravir by the HPLC method

A linear relationship was observed by plotting the drug concentration against the peak areas. Dolutegravir showed a linear response in the concentration range of 5-35 µg/mL by the HPLC method. The regression of the plot was computed by the least-squares regression method. Linearity results are presented in Table 3 and the linearity plot is shown in Figure 4.

**Tab. 3:**
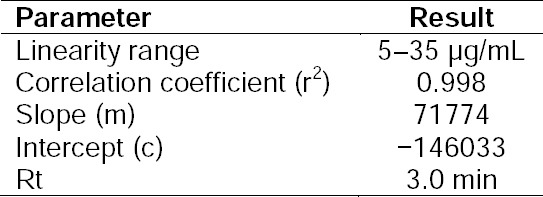
Linearity Data

#### Precision

Precision is the degree of repeatability of an analytical method under normal operational conditions. The developed method was found to be precise as the % RSD values for repeatability and intermediate precision studies were less than 2%. The results of repeatability and intermediate precision are shown in Table 4.

**Tab. 4:**
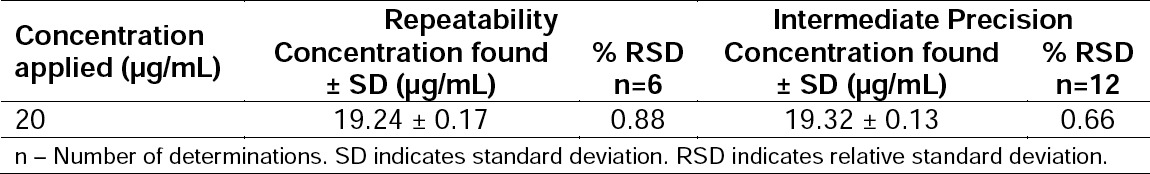
Results of Precision Studies by the HPLC Method

#### Accuracy

The results of the recovery studies showed the accuracy of the method. Satisfactory recoveries ranging from 98.82 to 99.70% were obtained by the proposed method. This indicated that the proposed method was accurate. The results obtained are given in Table 5.

**Tab. 5:**
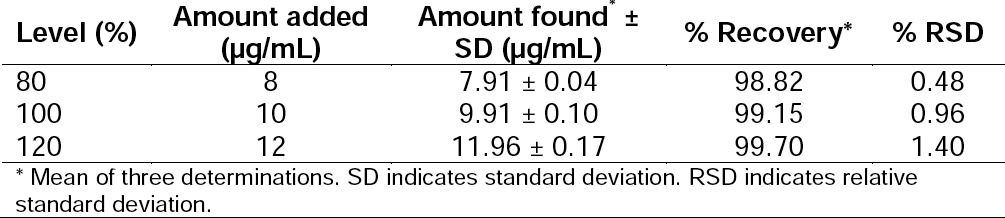
Results of Recovery Studies by the HPLC Method

#### Robustness

The robustness study was performed by a slight modification in the flow rate of the mobile phase and pH of the water. Samples of dolutegravir at 10 μg/mL concentration were analyzed under these changed experimental conditions. It was observed that there were no marked changes in chromatograms, which demonstrated that the developed method was robust in nature. The results are given in Table 6.

**Tab. 6:**
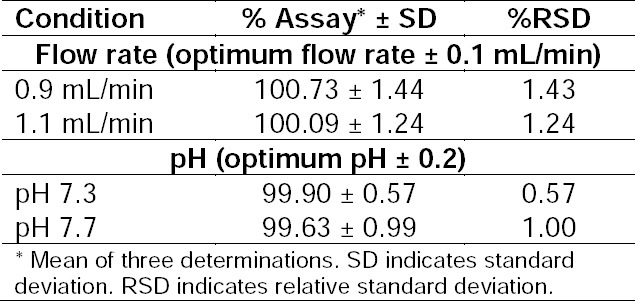
Results of Robustness Studies by the HPLC Method

#### Limit of Detection and Limit of Quantification

The LOD and LOQ were found to be 1.91 µg/mL and 5.17 µg/mL, respectively.

#### Specificity

**Fig. 5:**
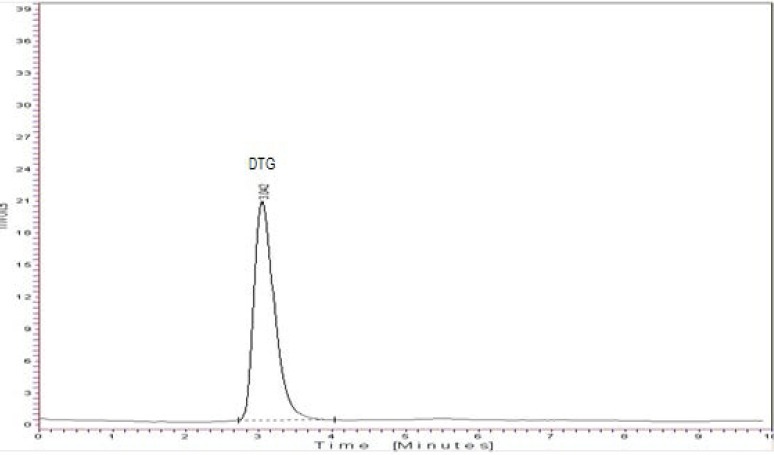
Typical chromatogram of dolutegravir sodium tablet sample

The specificity of method was performed by comparing the chromatograms of the blank, standard, and sample. It was found that there was no interference due to the excipient in the tablet formulation (Figure 5). A good correlation was also found between the retention time of the standard and sample of dolutegravir.

#### Solution Stability

The solution was found to be stable at ambient temperature for 24 h, and no unknown peaks were observed. The results of the stability studies by the HPLC method are shown in Table 7.

**Tab. 7:**
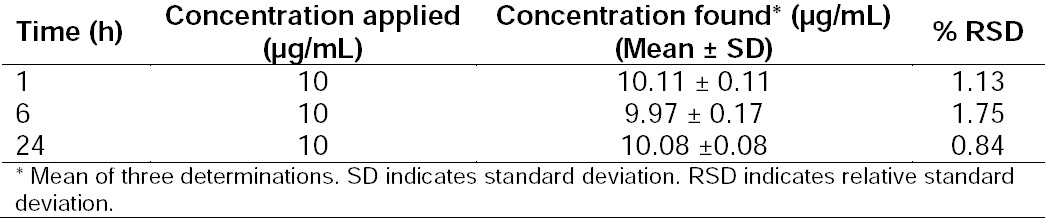
Results of Stability Studies by the HPLC Method

#### System Suitability

System suitability was studied by injecting six replicates of the standard solution. The system suitability parameters are given in Table 8. The number of theoretical plates was found to be 19944, which indicates efficient performance of the column.

**Tab. 8:**
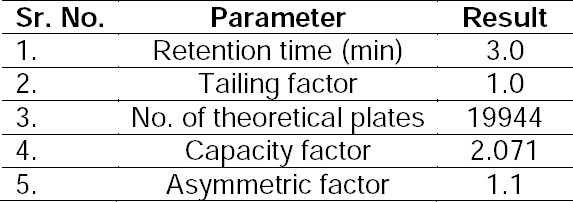
System Suitability Parameters by the HPLC Method

## Method B: HPTLC

### Analysis of the Pharmaceutical Dosage Form by the HPTLC method

A single spot at the Rf value of 0.77 was observed in the chromatogram of the drug samples extracted from the tablet. There was no interference from the excipients that are commonly present in the formulations. The drug content was found to be 101.62 % as given in Table 9.

**Tab. 9:**
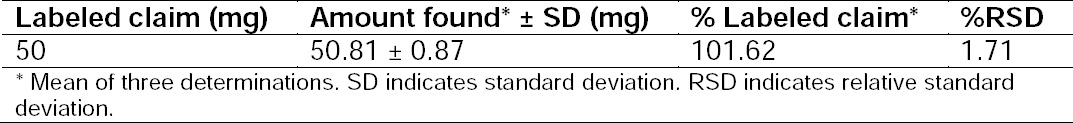
Analysis of Tablet Formulation by the HPTLC Method

### Method Validation

#### Linearity

**Fig. 6:**
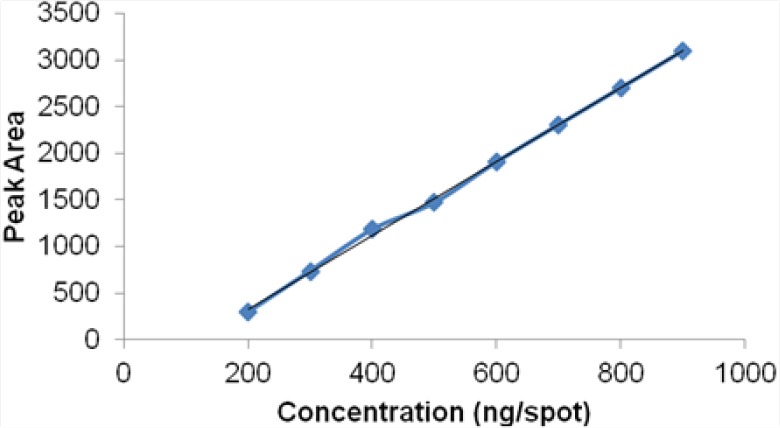
Linearity plot for dolutegravir by the HPTLC method

A linear relationship was observed by plotting the drug concentration against the peak areas. Dolutegravir showed a linear response in the concentration range of 200–900 ng/spot by the HPTLC method (Figure 6). The corresponding linear regression equation was Y = −468.1 + 3.966x with the square of the correlation co-effcient (*r*^2^) of 0.997 for dolutegravir as shown in Table 10.

**Tab. 10:**
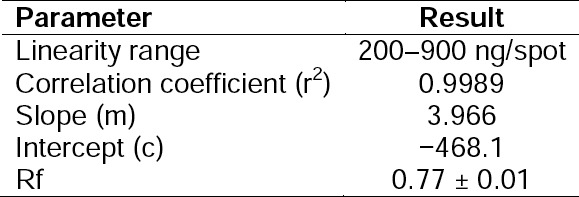
Linear Regression Data for the HPTLC Method

#### Precision

The results of the repeatability and intermediate precision experiments are shown in Table 11. The developed method was found to be precise as the % RSD values for repeatability and intermediate precision studies were < 2%, respectively.

**Tab. 11:**
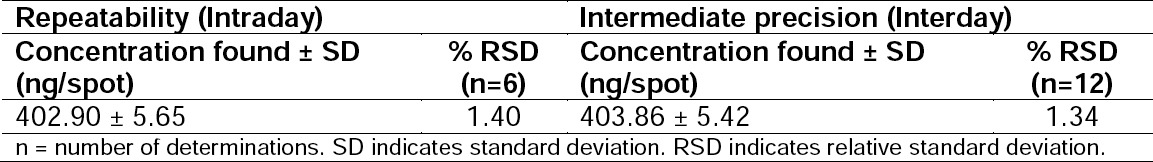
Results of Precision Studies by the HPTLC Method

#### Limit of Detection and Limit of Quantitation

The LOD and LOQ were found to be 28.61 ng/spot and 86.69 ng/spot, respectively.

#### Accuracy

The developed method showed high and consistent recoveries at all studied levels. The results obtained from the recovery studies are presented in Table 12. The mean % recovery ranged from 98.67% to 99.70%. Additionally, the obtained recoveries were found to be normally distributed with low % RSD at all concentration levels.

**Tab. 12:**
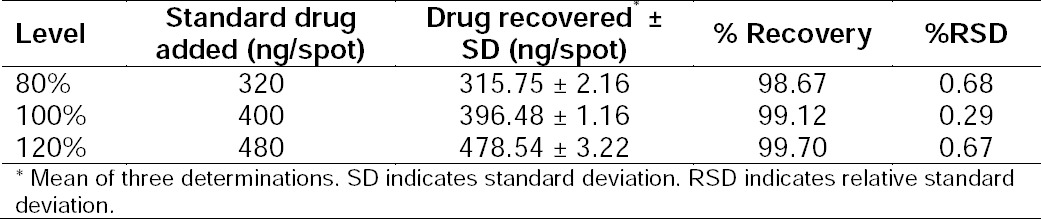
Results of Recovery Studies of Dolutegravir by the HPTLC Method

#### Specificity

**Fig. 7:**
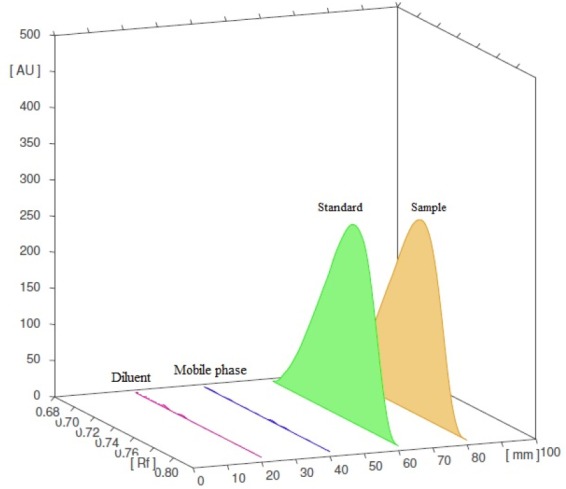
Densitogram indicating specificity of the HPTLC method for dolutegravir

**Fig. 8:**
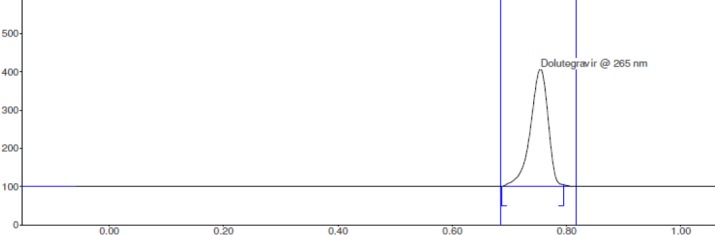
Densitogram of dolutegravir in tablet solution by the HPTLC method

The peak purity for dolutegravir was assessed by means of comparing the chromatogram of the sample with that of the mobile phase, diluents, and standard (Figure 7). No interference of excipients with the dolutegravir peak was observed in the sample chromatogram. A single peak of dolutegravir in the tablet solution was observed at Rf 0.77 (Figure 8).

#### Solution Stability

There was no indication of degradation in the sample solutions of dolutegravir as revealed by the peak purity data and from the value of % RSD (< 2%) for the peak areas of the bands of solution stored at different times. The solution was found to be stable at ambient temperature for 24 h, and no unknown peaks were observed. The results are shown in Table 13.

**Tab. 13:**
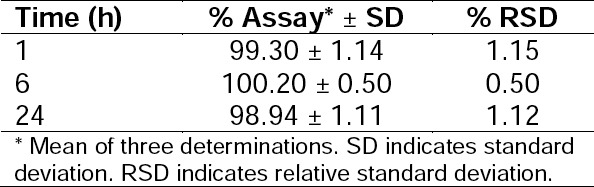
Results of Stability Studies

## Conclusion

A simple and reliable HPLC method has been developed and successfully validated for the estimation of dolutegravir in the presence of degradation products. As the method separates the drug from its degradation products, it can be employed as a stability-indicating one. The results of the HPLC and HPTLC validation tests indicated that the method was accurate, precise, robust, and reproducible. This assay system provides an accurate, precise, and sensitive method for dolutegravir quantitation and was successfully applied to the bulk and pharmaceutical dosage form. Hence, the proposed HPLC and HPTLC method is suitable for routine determination of dolutegravir in pharmaceutical formulation in quality control laboratories, where economy and time are essential.
